# The gene-expression profile of renal medulla in ISIAH rats with inherited stress-induced arterial hypertension

**DOI:** 10.1186/s12863-016-0462-6

**Published:** 2016-12-22

**Authors:** Marina A. Ryazanova, Larisa A. Fedoseeva, Nikita I. Ershov, Vadim M. Efimov, Arcady L. Markel, Olga E. Redina

**Affiliations:** 1 Institute of Cytology and Genetics, Siberian Branch of Russian Academy of Sciences, Novosibirsk, Russian Federation; 2 Novosibirsk State University, Novosibirsk, Russian Federation

**Keywords:** Stress-sensitive hypertension, Renal medulla, Transcriptional profiling, RNA-Seq, ISIAH rats

## Abstract

**Background:**

The changes in the renal function leading to a reduction of medullary blood flow can have a great impact on sodium and water homeostasis and on the long-term control of arterial blood pressure. The RNA-Seq approach was used for transcriptome profiling of the renal medulla from hypertensive ISIAH and normotensive WAG rats to uncover the genetic basis of the changes underlying the renal medulla function in the ISIAH rats being a model of the stress-sensitive arterial hypertension and to reveal the genes which possibly may contribute to the alterations in medullary blood flow.

**Results:**

Multiple DEGs specifying the function of renal medulla in ISIAH rats were revealed. The group of DEGs described by Gene Ontology term ‘oxidation reduction’ was the most significantly enriched one. The other groups of DEGs related to response to external stimulus, response to hormone (endogenous) stimulus, response to stress, and homeostatic process provide the molecular basis for integrated responses to homeostasis disturbances in the renal medulla of the ISIAH rats. Several DEGs, which may modulate the renal medulla blood flow, were detected. The reduced transcription of *Nos3* pointed to the possible reduction of the blood flow in the renal medulla of ISIAH rats.

**Conclusions:**

The generated data may be useful for comparison with those from different models of hypertension and for identifying the common molecular determinants contributing to disease manifestation, which may be potentially used as new pharmacological targets.

**Electronic supplementary material:**

The online version of this article (doi:10.1186/s12863-016-0462-6) contains supplementary material, which is available to authorized users.

## Background

The study of molecular-genetic mechanisms of hypertension is an important task for biology and medicine. Multifactorial etiology of hypertension complicates the solution of this problem, and despite of numerous studies, the basal molecular mechanisms of essential hypertension remain not fully elucidated. However, it has long been known that renal dysfunction underlies the development of all forms of hypertension in experimental animals and humans [1–4].

The ISIAH (Inherited Stress-Induced Arterial Hypertension) rat strain was developed to study the genetic background of the stress-sensitive form of arterial hypertension and its complications. Selection of the ISIAH rats from an outbred Wistar stock was performed for a systolic arterial blood pressure (BP) elevation induced by 0.5 h emotional stress, which was caused by keeping the rats restricted in a small wire mesh cage. The ISIAH rats acquire the elevated basal systolic arterial BP at the age of 6 weeks (175.0 ± 3.5 mmHg in males and 165.0 ± 3.0 mmHg in females) and systolic arterial BP in these rats dramatically increases under the restriction conditions [5, 6]. The development of the hypertensive state in ISIAH rats is also accompanied by a hypertrophy of the left ventricle, increase in the wall thickness of the small arteries, and changes in the electrocardiographic pattern [6]. The studies on the kidney histology showed the alterations, which were indicative of an increase in filtration barrier functional load and of processes leading to the development of glomerular [7] and medullary sclerosis in ISIAH rats [8].

The unraveling of the genetic basis of the renal function in ISIAH rats may be useful for understanding the mechanisms underlying the stress-sensitive hypertension development and for identifying the molecular determinants, which may be potentially used as the therapeutic targets for pharmacological intervention.

Recently, using the next-generation RNA sequencing (RNA-Seq) approach, we analyzed the renal cortex transcriptome in ISIAH rats [9]. The results of this study showed that the functioning of the renal cortex in ISIAH rats is based on the changes in transcriptional activity of multiple genes related to different biological processes and metabolic pathways. However, in a number of studies it was demonstrated that the initial changes leading to the pathology of renal function begin from medullary blood flow reduction, and these changes may exert significant effects on sodium and water homeostasis and on the long-term control of arterial BP [2, 10, 11].

Thus, the goal of the current study was to uncover the genetic basis of the changes underlying the renal medulla function in the ISIAH rats and to reveal the features which possibly may be related to the alterations in medullary blood flow. To achieve this goal the RNA-Seq approach was used for transcriptome profiling of the renal medulla from hypertensive ISIAH and normotensive WAG rats. The differentially expressed genes (DEGs) related to stress-sensitive hypertension and possibly to the alterations in medullary blood flow as well as the metabolic pathways contributing to the inter-strain differences in renal medulla functions were detected. Several DEGs, which may modulate the renal medulla blood flow, were identified and their possible impact on the process was discussed.

## Results

The expression of 13,646 genes was detected in the renal medulla of analyzed rats. The comparative analysis of their expression in the renal medulla of ISIAH and WAG rats revealed 960 DEGs (listed in the Additional file 1). The Additional file 2 represents the heatmap for the DEGs. Approximately a half of these genes (524 genes, i.e., 54.7%) were down-regulated in the renal medulla of ISIAH rats. The expression of 11 genes was detected in renal medulla of only one rat strain (Additional file 3). One of these genes (*Retn,* resistin) is known as associated with hypertension. Its expression was detected in renal medulla of ISIAH rats but not in WAG. The list of the top 40 genes with the highest differences in their expression in renal medulla of ISIAH and WAG rats included two genes (*Acsm3,* acyl-CoA synthetase medium-chain family member 3; and *Ephx2,* epoxide hydrolase 2, cytoplasmic), which were annotated in Rat Genome Database (RGD) as genes related to hypertension (Table 1).

**Table 1:** Top 40 genes with the greatest difference in expression between ISIAH and WAG renal medulla

Gene symbol	NCBI gene ID	Gene definition	log2 (fold_change) ISIAH/WAG
*LOC102546948*	102546948	uncharacterized LOC102546948	−5.73
*Galnt13*	311039	UDP-N-acetyl-alpha-D-galactosamine:polypeptide N-acetylgalactosaminyltransferase 13 (GalNAc-T13)	−5.56
*Pdilt*	293544	protein disulfide isomerase-like, testis expressed	−5.17
*LOC501110*	501110	similar to Glutathione S-transferase A1 (GTH1) (HA subunit 1) (GST-epsilon) (GSTA1-1) (GST class-alpha)	−4.59
*Slc10a2*	29500	solute carrier family 10 (sodium/bile acid cotransporter), member 2	−4.40
*Pcdh9*	306091	protocadherin 9	−4.23
*LOC100909561*	100909561	nuclease-sensitive element-binding protein 1-like	−3.74
*Car5a*	54233	carbonic anhydrase 5a, mitochondrial	−3.74
*LOC100361907*	100361907	complement factor H-related protein B	−3.73
*Sphkap*	316561	SPHK1 interactor, AKAP domain containing	−3.65
*LOC102552001*	102552001	uncharacterized LOC102552001	−3.62
*LOC102550987*	102550987	uncharacterized LOC102550987	−3.57
*Kcnj5*	29713	potassium inwardly-rectifying channel, subfamily J, member 5	−3.23
*Upk2*	689093	uroplakin 2	3.12
*LOC102555352*	102555352	uncharacterized LOC102555352	3.13
*LOC102551856*	102551856	uncharacterized LOC102551856	3.21
*Spta1*	289257	spectrin, alpha, erythrocytic 1 (elliptocytosis 2)	3.28
*LOC686967*	686967	similar to olfactory receptor 1442	3.34
*Acsm3* ^a^	24763	acyl-CoA synthetase medium-chain family member 3	3.40
*LOC100911960*	100911960	UDP-glucuronosyltransferase 1-9-like	3.44
*Thrsp*	25357	thyroid hormone responsive	3.51
*Sprr1a*	499660	small proline-rich protein 1A	3.51
*Hpgd*	79242	hydroxyprostaglandin dehydrogenase 15 (NAD)	3.52
*LOC102553290*	102553290	collagen alpha-1(III) chain-like	3.62
*Ly6al*	362935	lymphocyte antigen 6 complex, locus A-like	3.65
*Akr1b8*	286921	aldo-keto reductase family 1, member B8	3.70
*Shisa3*	498356	shisa family member 3	3.83
*Nefh*	24587	neurofilament, heavy polypeptide	3.90
*Tcerg1l*	361669	transcription elongation regulator 1-like	4.09
*Krt19*	360626	keratin 19	4.18
*Ephx2* ^a^	65030	epoxide hydrolase 2, cytoplasmic	4.52
*Ubd*	29168	ubiquitin D	4.61
*Resp18*	50561	regulated endocrine-specific protein 18	4.65
*Serpinb12*	304692	serpin peptidase inhibitor, clade B (ovalbumin), member 12	5.38
*Car3*	54232	carbonic anhydrase 3	5.54
*Fam111a*	499322	family with sequence similarity 111, member A	5.86
*LOC100362069*	100362069	ribosomal protein L28-like	6.11
*Gys2*	25623	glycogen synthase 2	6.38
*Stk32c*	365381	serine/threonine kinase 32C	6.41
*RGD1565131*	498143	60S ribosomal protein L15-like	8.94

^a^Genes are annotated in Rat Genome Database (http://rgd.mcw.edu/) as associated with hypertension. ISIAH and WAG – rat strains used in the study

**Fig. 1:**
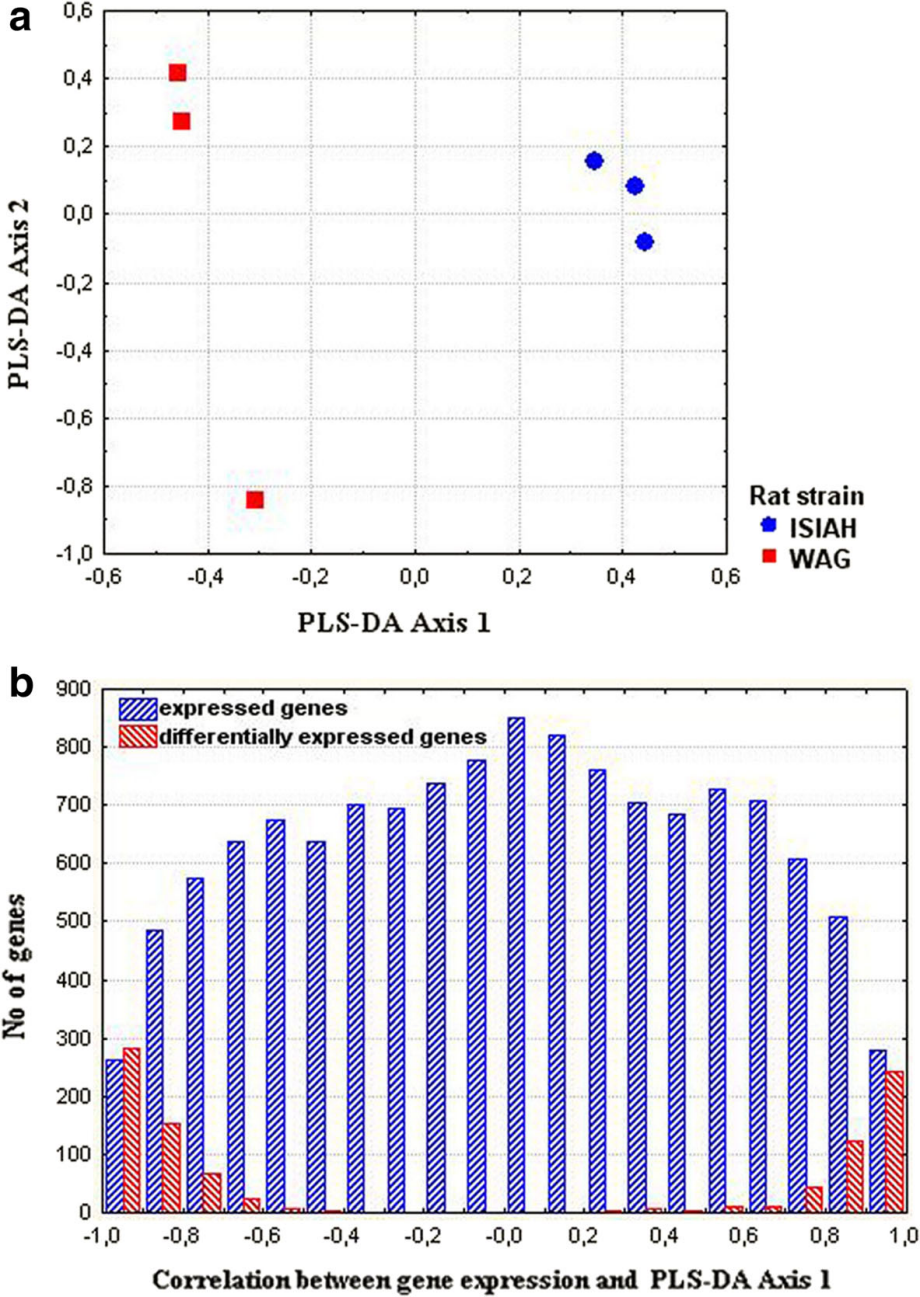
**a** Axes maximizing the distances between ISIAH and WAG rats. **b** The distribution of expressed genes along the first axis. The data are based on the Pearson correlation coefficients between the first axis and the level of genes expression

**Fig. 2:**
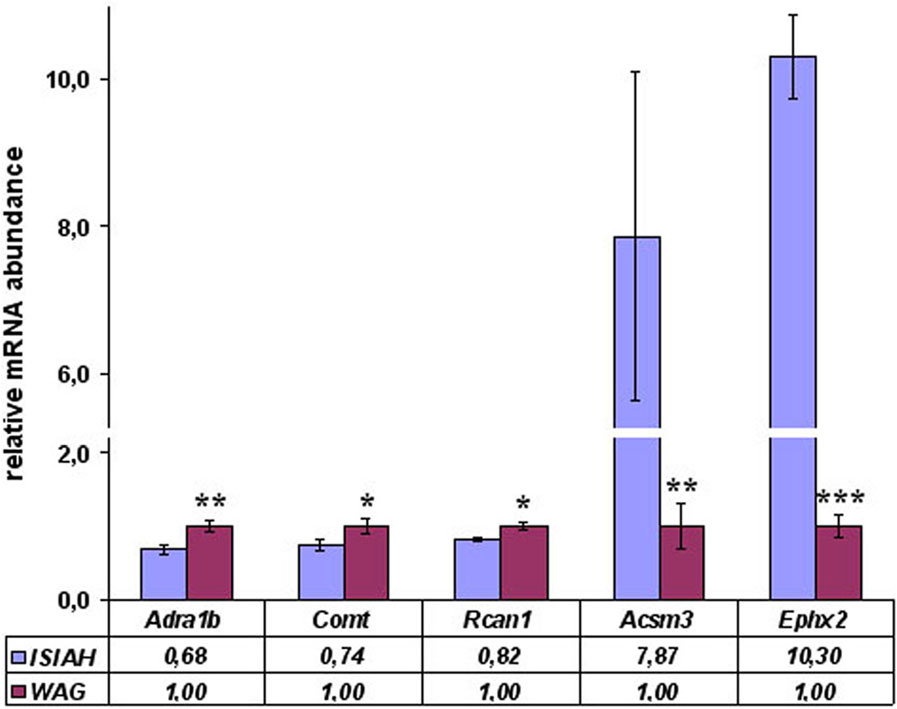
The relative mRNA abundance measured by qPCR. The normalized mRNA level in control samples of the WAG rats was assigned a value of 1. *Vertical bars* show the standard error of the mean, and significance of inter-strain difference is indicated by *p < 0.05, **p < 0.01, ***p < 0.001

**Fig. 3:**
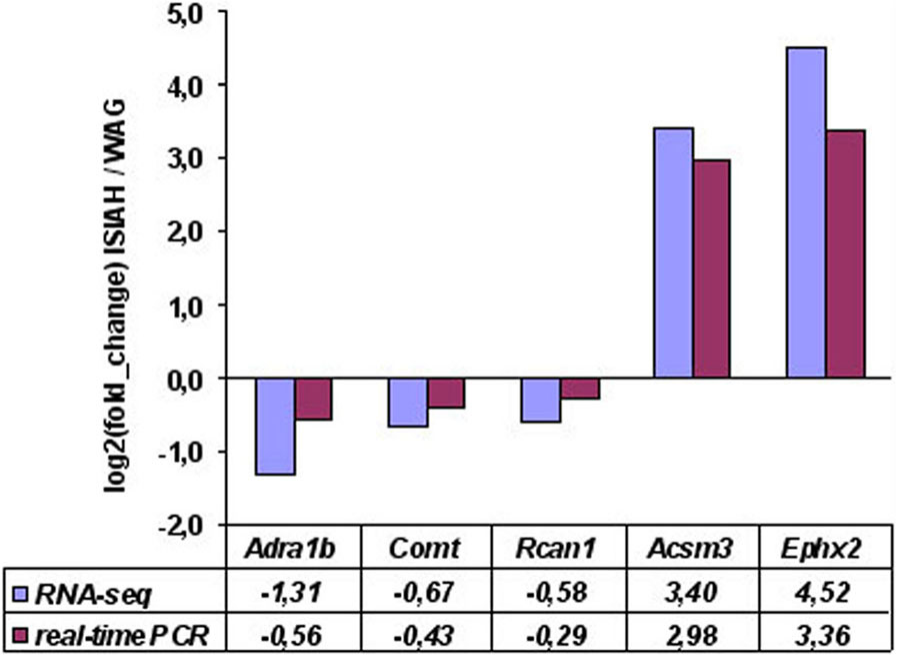
Comparison of gene expression level measurements obtained by RNA-seq and qPCR

The genes making the strongest contribution to the inter-strain differences were detected by the partial-least squares discriminant analysis (PLS-DA). The distances between ISIAH and WAG rats are shown in Fig. 1a, and the results of the correlation analysis between gene expression and PLS-DA Axis 1 are presented in Fig. 1b. The DEGs are shown in red in Fig. 1b. The DEGs in the most polar position contribute the most to the inter-strain variations. The correlation coefficients for *Acsm3* and *Ephx2* were 0.996 and 0.994, correspondingly. Hence, *Acsm3* and *Ephx2* may be considered as genes making the strongest contribution to the inter-strain differences. The differential expression of these and several other genes was validated by real-time PCR (Fig. 2). The correlation between the gene expression estimated by two methods (RNA-Seq and real-time PCR) was 0.99 (Fig. 3).

According to the RGD annotations, 58 DEGs found in the current study are associated with hypertension (Table 2). Seven of them (*Agtr1a, Fn1, Gja1, Lama2, Mmp2, Mmp9, Nos3*) are known as being associated with renal hypertension. The functional annotation in Database for Annotation, Visualization and Integrated Discovery (DAVID) additionally revealed four DEGs (*Col1a2, Guca2b, P2rx4, Pcsk5*) related to BP regulation. The transcription of most of genes associated with hypertension (67.2%) was reduced in the renal medulla of ISIAH rats. About a half of the DEGs related to hypertension are also known as associated with diabetic nephropathy and insulin resistance, and two DEGs were found to be related to nephrosclerosis (Table 2). Many of DEGs listed in the Table 2 are related to the diseases of immune system. The study revealed 76 DEGs referred to in RGD as associated with different renal diseases, such as renal insufficiency, nephrosclerosis, diabetic nephropathy, and renal fibrosis (Table 3).

**Table 2:** Genes differentially expressed in ISIAH and WAG renal medulla and referred to in Databases as associated with hypertension and blood pressure regulation

Gene symbol	NCBI gene ID	Gene name	log2 fold_change ISIAH/WAG
Rat Genome Database			
*Acsm3* ^e^	24763	acyl-CoA synthetase medium-chain family member 3	3.40
*Adipoq* ^c,d,e^	246253	adiponectin, C1Q and collagen domain containing	1.17
*Adra1b* ^c^	24173	adrenoceptor alpha 1B	−1.31
*Adra2a*	25083	adrenoceptor alpha 2°	−1.15
*Agtr1a* ^a,c,d,e^	24180	angiotensin II receptor, type 1°	−0.47
*Alas1*	65155	aminolevulinate, delta-, synthase 1	0.74
*Angpt2* ^e^	89805	angiopoietin 2	−0.79
*Aqp2* ^d^	25386	aquaporin 2 (collecting duct)	−0.47
*Aqp4* ^e^	25293	aquaporin 4	−0.59
*Clu* ^d,e^	24854	clusterin, transcript variant X1	−0.80
*Cnr1* ^c^	25248	cannabinoid receptor 1 (brain)	−0.58
*Comt*	24267	catechol-O-methyltransferase	−0.67
*Corin*	289596	corin, serine peptidase	−1.04
*Cst3* ^c,d,e^	25307	cystatin C	−0.50
*Cyp1a1* ^e^	24296	cytochrome P450, family 1, subfamily a, polypeptide 1	−0.51
*Cyp4f1*	56266	cytochrome P450, family 4, subfamily f, polypeptide 1	0.60
*Dio2* ^c^	65162	deiodinase, iodothyronine, type II	−2.56
*Dusp1* ^e^	114856	dual specificity phosphatase 1	−0.71
*Ebag9*	299864	estrogen receptor binding site associated, antigen, 9	0.49
*Emilin1*	298845	elastin microfibril interfacer 1	−0.65
*Ephx1* ^e^	25315	epoxide hydrolase 1, microsomal (xenobiotic)	0.70
*Ephx2* ^c,d^	65030	epoxide hydrolase 2, cytoplasmic	4.52
*F2* ^d, e^	29251	coagulation factor II	−0.93
*Fbn1* ^d^	83727	fibrillin 1, transcript variant X1	−0.61
*Fn1* ^a,d,e^	25661	fibronectin 1	−0.98
*Gja1* ^a,e^	24392	gap junction protein, alpha 1	0.99
*Gstm2*	24424	glutathione S-transferase mu 2	−0.76
*Gstp1* ^e^	24426	glutathione S-transferase pi 1	0.53
*Hgf* ^c,d,e^	24446	hepatocyte growth factor	−0.77
*Hsd17b4* ^e^	79244	hydroxysteroid (17-beta) dehydrogenase 4	0.52
*Igf1* ^d,e^	24482	insulin-like growth factor 1	−0.75
*Itgav* ^c^	296456	integrin, alpha V	−0.60
*Lama2* ^a^	309368	laminin, alpha 2	−0.58
*Lox*	24914	lysyl oxidase	−0.81
*Mif* ^c,e^	81683	macrophage migration inhibitory factor (glycosylation-inhibiting factor)	0.55
*Mmp2* ^a,e^	81686	matrix metallopeptidase 2	−0.54
*Mmp9* ^ad,e^	81687	matrix metallopeptidase 9	−2.00
*Mthfr* ^b,d,e^	362657	methylenetetrahydrofolate reductase (NAD(P)H)	0.49
*Nos3* ^a,c,d,e^	24600	nitric oxide synthase 3, endothelial cell	−0.55
*Nox4* ^d^	85431	NADPH oxidase 4	0.50
*Pappa*	313262	pregnancy-associated plasma protein A	−0.68
*Pecam1* ^d,e^	29583	platelet/endothelial cell adhesion molecule 1	−0.45
*Ptgds* ^b,c,d^	25526	prostaglandin D2 synthase (brain)	−0.78
*Ptk2b* ^c,e^	50646	protein tyrosine kinase 2 beta	−0.79
*Retn* ^c,d,e^	246250	resistin	expression was detected only in ISIAH rats
*Rnpep*	81761	arginyl aminopeptidase (aminopeptidase B)	−0.47
*RT1-Bb* ^e^	309622	RT1 class II, locus Bb	−1.26
*Serpine2*	29366	serpin peptidase inhibitor, clade E (nexin, plasminogen activator inhibitor type 1), member 2	−0.55
*Slc26a4*	29440	solute carrier family 26 (anion exchanger), member 4	0.59
*Slc2a4* ^c^	25139	solute carrier family 2 (facilitated glucose transporter), member 4	−0.76
*Slc5a2*	64522	solute carrier family 5 (sodium/glucose cotransporter), member 2	0.64
*Slc6a19*	664630	solute carrier family 6 (neutral amino acid transporter), member 19	0.48
*Slc8a1*	29715	solute carrier family 8 (sodium/calcium exchanger), member 1	0.53
*Slc9a3r2*	116501	solute carrier family 9, subfamily A (NHE3, cation proton antiporter 3), member 3 regulator 2	−0.49
*Sod3* ^c,d^	25352	superoxide dismutase 3, extracellular	0.56
*Tacr3*	24808	tachykinin receptor 3	−1.22
*Tf* ^c,d,e^	24825	transferrin	−1.43
*Vcam1* ^c,d,e^	25361	vascular cell adhesion molecule 1	−0.86
DAVID			
*Col1a2* ^e^	84352	collagen, type I, alpha 2	−0.53
*Guca2b*	64055	guanylate cyclase activator 2B	2.03
*P2rx4*	29659	purinergic receptor P2X, ligand-gated ion channel 4	−1.41
*Pcsk5*	116548	proprotein convertase subtilisin/kexin type 5	0.73

ISIAH and WAG – rat strains used in the study. *DAVID* – Database for Annotation, Visualization and Integrated Discovery (http://david.abcc.ncifcrf.gov/)

Genes associated with: ^a^renal hypertension; ^b^nephrosclerosis; ^c^insulin resistance; ^d^diabetic nephropathy; ^e^immune system diseases

**Table 3:** Genes differentially expressed in ISIAH versus WAG renal medulla and annotated in Rat Genome Database as associated with kidney diseases

Gene symbol	NCBI gene ID	Gene name	log2 fold change ISIAH/WAG
*Acsm3*	24763	acyl-CoA synthetase medium-chain family member 3	3.40
*Adipoq* ^c,d^	246253	adiponectin, C1Q and collagen domain containing	1.17
*Agtr1a* ^a,c,d^	24180	angiotensin II receptor, type 1a	−0.47
*Ak4* ^c^	29223	adenylate kinase 4	0.63
*Alpl*	25586	alkaline phosphatase, liver/bone/kidney	0.53
*Amacr*	25284	alpha-methylacyl-CoA racemase	0.50
*Angpt2* ^a^	89805	angiopoietin 2	−0.79
*Anxa2*	56611	annexin A2	−0.45
*Apoh* ^d^	287774	apolipoprotein H (beta-2-glycoprotein I)	−1.52
*Aqp2* ^d^	25386	aquaporin 2 (collecting duct)	−0.47
*Baat*	29725	bile acid CoA: amino acid N-acyltransferase (glycine N-choloyltransferase)	0.78
*Car2*	54231	carbonic anhydrase 2	0.53
*Cftr*	24255	cystic fibrosis transmembrane conductance regulator	−1.22
*Cldn19*	298487	claudin 19	−0.53
*Clu* ^a,c,d^	24854	clusterin	−0.80
*Col3a1* ^a,c^	84032	collagen, type III, alpha 1	−0.64
*Col4a5*	363457	collagen, type IV, alpha 5	−0.45
*Comt*	24267	catechol-O-methyltransferase	−0.67
*Csf1r* ^c,d^	307403	colony stimulating factor 1 receptor	−0.73
*Cst3* ^d^	25307	cystatin C	−0.50
*Cubn* ^d^	80848	cubilin (intrinsic factor-cobalamin receptor)	0.68
*Cyp1a1* ^c^	24296	cytochrome P450, family 1, subfamily a, polypeptide 1	−0.51
*Cyp4a3*	298423	cytochrome P450, family 4, subfamily a, polypeptide 3	0.52
*Dusp1*	114856	dual specificity phosphatase 1	−0.71
*Ebag9*	299864	estrogen receptor binding site associated, antigen, 9	0.49
*Ephx2* ^c,d^	65030	epoxide hydrolase 2, cytoplasmic	4.52
*F2* ^c,d^	29251	coagulation factor II	−0.93
*Fbn1* ^a,d^	83727	fibrillin 1	−0.61
*Fga* ^c^	361969	fibrinogen alpha chain	0.85
*Fhit*	60398	fragile histidine triad	1.58
*Fmod* ^d^	64507	fibromodulin	−1.09
*Fn1* ^a,c,d^	25661	fibronectin 1	−0.98
*Gatm* ^c^	81660	glycine amidinotransferase (L-arginine:glycine amidinotransferase)	0.86
*Gja1*	24392	gap junction protein, alpha 1	0.99
*Gpc1*	58920	glypican 1	−0.72
*Gstp1* ^c^	24426	glutathione S-transferase pi 1	0.53
*Gtpbp4* ^c^	114300	GTP binding protein 4	3.01
*Hao1*	311446	hydroxyacid oxidase (glycolate oxidase) 1	−1.60
*Hgf* ^d^	24446	hepatocyte growth factor	−0.77
*Igf1* ^d^	24482	insulin-like growth factor 1	−0.75
*Igfbp1* ^d^	25685	insulin-like growth factor binding protein 1	1.26
*Il4r*	25084	interleukin 4 receptor	0.64
*Lama2*	309368	laminin, alpha 2	−0.58
*Lgals1*	56646	lectin, galactoside-binding, soluble, 1	0.88
*Lrp1* ^a^	299858	low density lipoprotein receptor-related protein 1	−0.53
*Lrp5*	293649	low density lipoprotein receptor-related protein 5	−0.47
*Ltbp1*	59107	latent transforming growth factor beta binding protein 1	−0.57
*Mif*	81683	macrophage migration inhibitory factor (glycosylation-inhibiting factor)	0.55
*Mme*	24590	membrane metallo-endopeptidase	1.35
*Mmp2* ^a, c^	81686	matrix metallopeptidase 2	−0.54
*Mmp9* ^a, d^	81687	matrix metallopeptidase 9	−2.00
*Mok*	362787	MOK protein kinase	−0.93
*Mthfr* ^b*,* c, d^	362657	methylenetetrahydrofolate reductase (NAD(P)H)	0.49
*Nos3* ^c, d^	24600	nitric oxide synthase 3, endothelial cell	−0.55
*Nox4* ^d^	85431	NADPH oxidase 4	0.50
*Nphs2* ^a^	170672	nephrosis 2, idiopathic, steroid-resistant	0.57
*Pecam1* ^d^	29583	platelet/endothelial cell adhesion molecule 1	−0.45
*Pla2g4a*	24653	phospholipase A2, group IVA (cytosolic, calcium-dependent)	−0.69
*Ptgds* ^b, d^	25526	prostaglandin D2 synthase (brain)	−0.78
*Ptk2b*	50646	protein tyrosine kinase 2 beta	−0.79
*Rap1gap* ^a^	313644	Rap1 GTPase-activating protein	−0.60
*Retn* ^c, d^	246250	resistin	expression was detected only in ISIAH rats
*RT1-Bb*	309622	RT1 class II, locus Bb	−1.26
*Serpinf1* ^d^	287526	serpin peptidase inhibitor, clade F (alpha-2 antiplasmin, pigment epithelium derived factor), member 1	−2.10
*Serping1*	295703	serpin peptidase inhibitor, clade G (C1 inhibitor), member 1	0.56
*Sfrp1*	84402	secreted frizzled-related protein 1	−0.61
*Slc17a2*	306950	solute carrier family 17, member 2	0.59
*Slc19a3* ^c^	316559	solute carrier family 19 (thiamine transporter), member 3	1.00
*Slc5a2*	64522	solute carrier family 5 (sodium/glucose cotransporter), member 2	0.64
*Slc6a19*	664630	solute carrier family 6 (neutral amino acid transporter), member 19	0.48
*Slit2*	360272	slit homolog 2 (Drosophila)	0.45
*Sod3* ^d^	25352	superoxide dismutase 3, extracellular	0.56
*Tf* ^d^	24825	transferrin	−1.43
*Timp2* ^d^	29543	TIMP metallopeptidase inhibitor 2	−0.52
*Vcam1* ^c,d^	25361	vascular cell adhesion molecule 1	−0.86
*Vtn* ^d^	29169	vitronectin	−1.18

Genes associated with: ^a^renal fibrosis; ^b^nephrosclerosis; ^c^renal insufficiency, ^d^diabetic nephropathy; ISIAH and WAG – rat strains used in the study

The differential transcription of 59 transcription factor genes was found in the renal medulla of ISIAH and WAG rats (Table 4). No one of these genes is currently referred to in databases as associated with arterial hypertension or kidney diseases.

**Table 4:** Transcription factor genes differentially expressed in ISIAH and WAG renal medulla

Gene symbol	NCBI gene ID	Gene name	log2 fold_change ISIAH/WAG
*Alx1*	25401	ALX homeobox 1	−1.13
*Arntl*	29657	aryl hydrocarbon receptor nuclear translocator-like	−1.01
*Bcl6*	303836	B-cell CLL/lymphoma 6	−2.02
*Bcl6b*	360551	B-cell CLL/lymphoma 6, member B	−0.80
*Bhlhe22*	365748	basic helix-loop-helix family, member e22	−1.08
*Bnc2*	298189	basonuclin 2	0.61
*Btbd11*	314675	BTB (POZ) domain containing 11	−0.65
*Ccrn4l*	310395	CCR4 carbon catabolite repression 4-like (S, cerevisiae)	0.64
*Cebpd*	25695	CCAAT/enhancer binding protein (C/EBP), delta	−0.83
*Dab2*	79128	disabled 2, mitogen-responsive phosphoprotein	0.49
*Dmrt2*	309430	doublesex and mab-3 related transcription factor 2	−0.55
*Esrrb*	299210	estrogen-related receptor beta	−0.58
*Etv5*	303828	ets variant 5	−0.70
*Fhl1*	25177	four and a half LIM domains 1	1.09
*Gcfc2*	312474	GC-rich sequence DNA-binding factor 2	−0.61
*Gne*	114711	glucosamine (UDP-N-acetyl)-2-epimerase/N-acetylmannosamine kinase	−0.51
*Grhl1*	313993	grainyhead-like 1 (Drosophila)	1.91
*Hand2*	64637	heart and neural crest derivatives expressed 2	expression was detected only in ISIAH rats
*Hes6*	316626	hes family bHLH transcription factor 6	−0.72
*Id3*	25585	inhibitor of DNA binding 3	−0.50
*Id4*	291023	inhibitor of DNA binding 4	0.47
*Ilf2*	310612	interleukin enhancer binding factor 2	−0.46
*Irf7*	293624	interferon regulatory factor 7	0.59
*Irf8*	292060	interferon regulatory factor 8	−0.58
*Khdrbs3*	64015	KH domain containing, RNA binding, signal transduction associated 3	−1.23
*Lmo2*	362176	LIM domain only 2	−0.79
*Map2*	25595	microtubule-associated protein 2	−1.44
*Mcm7*	288532	minichromosome maintenance complex component 7	0.61
*Mef2c*	499497	myocyte enhancer factor 2C	−0.59
*Mybl1*	297783	myeloblastosis oncogene-like 1	−0.56
*Nfkbil1*	361794	nuclear factor of kappa light polypeptide gene enhancer in B-cells inhibitor-like 1	0.98
*Notch3*	56761	notch 3	−0.50
*Npas2*	316351	neuronal PAS domain protein 2	−0.90
*Nr2f1*	81808	nuclear receptor subfamily 2, group F, member 1	−1.31
*Nrip3*	361625	nuclear receptor interacting protein 3	−0.83
*Osr1*	298878	odd-skipped related transciption factor 1	−1.29
*Osr2*	315039	odd-skipped related transciption factor 2	1.17
*P8*	113900	nuclear proten 1	−0.95
*Patz1*	305471	POZ (BTB) and AT hook containing zinc finger 1	0.57
*Paxbp1*	681004	PAX3 and PAX7 binding protein 1	−0.53
*Pou2af1*	690528	POU class 2 associating factor 1	1.19
*Prdm6*	307305	PR domain containing 6	1.50
*Prox1*	305066	prospero homeobox 1	−1.06
*Sim1*	309888	single-minded family bHLH transcription factor 1	−0.50
*Smarcd3*	296732	SWI/SNF related, matrix associated, actin dependent regulator of chromatin, subfamily d, member 3	−0.58
*Spry4*	291610	sprouty homolog 4 (Drosophila)	−0.61
*Stat5a*	24918	signal transducer and activator of transcription 5A	0.63
*Tcerg1l*	361669	transcription elongation regulator 1-like	4.09
*Tcf21*	252856	transcription factor 21	−0.48
*Tcf4*	84382	transcription factor 4	−0.66
*Tle2*	299636	transducin-like enhancer of split 2 (E(sp1) homolog, Drosophila)	−0.61
*Zbtb16*	353227	zinc finger and BTB domain containing 16	−1.11
*Zdhhc2*	246326	zinc finger, DHHC-type containing 2	0.75
*Zfhx2*	305888	zinc finger homeobox 2	−0.88
*Zfp189*	313219	zinc finger protein 189	−0.62
*Zfp354a*	24522	zinc finger protein 354A	0.58
*Zfp449*	684901	zinc finger protein 449	−0.71
*Zfp710*	293044	zinc finger protein 710	−0.76
*Zfp958*	100302405	zinc finger protein 958	−0.64

ISIAH and WAG – rat strains used in the study

Gene Ontology (GO) terms for biological processes defined as significantly enriched within the analysis in DAVID are represented in Additional file 4. GO terms which might be essential for stress-sensitive hypertension development in ISIAH rats are shown in bold in this file, and the details for DEGs from these groups are given in Additional file 5.

The most significantly enriched GO terms were those related to oxidation reduction, lipid metabolic process, response to external stimulus as well as regulation of response to external stimuli. The analysis showed that the differences in renal medulla function between ISIAH and WAG rats are under control of many genes participating in response to hormone stimulus including genes associated with response to steroid (glucocorticoid) hormone stimulus.

Multiple DEGs were related to transport, (including sodium ion transport, water transport, glucose transport, lipid transport) and regulation of transport. Many DEGs associated with transport are annotated also as responsible for homeostatic process, including ion homeostasis.

Several GO terms were related to BP control. These were the groups of DEGs associated with blood circulation, regulation of BP, and negative regulation of blood coagulation.

Two large groups of DEGs were associated with response to stress and regulation of response to stress. The specificity of the stress types was defined by GO terms related to responses to oxidative stress, to osmotic stress, and to salt stress.

The other processes which might be involved in hypertension development in ISIAH rats were: neurogenesis, regulation of action potential in neuron, angiogenesis, regulation of smooth muscle cell differentiation, cell adhesion and regulation of cell adhesion.

The functional annotation underlined the important role of the immune system process and its regulation. The analysis in the Kyoto Encyclopedia of Genes and Genomes Pathway Database (KEGG) performed within the DAVID highlighted sixteen pathways that were significantly (p < 0.05) enriched (Additional file 6). Several of them were also related to immune system functioning. All of the pathways contained genes annotated in RGD as associated with hypertension or with kidney diseases.

## Discussion

The transcriptome profiling of the renal medulla from ISIAH and WAG rats let to identify multiple DEGs and several metabolic pathways contributing to differences between the renal medulla functions in ISIAH rats with stress-sensitive hypertension and normotensive controls. The functional annotation of DEGs within the analysis in Databases demonstrated that many of them are associated with hypertension and regulation of BP. One of these, *Retn*, was expressed in renal medulla of hypertensive rats but not in controls. However, the low level of expression of this gene was reported in kidney from Fischer 344 male rats, too [12]. So, the inter-strain differences in transcriptional activity of *Retn* shouldn’t be essential for hypertension development in ISIAH rats.

In recent years, it has become clear that a key determinant of the set point of the renal pressure-natriuresis curve is the balance of reactive oxygen and nitrogen species within the renal medullary region [11]. The experimentally induced elevations of either superoxide or hydrogen peroxide in the renal medulla result in reduction of medullary blood flow, enhanced sodium reabsorption, and hypertension [13].

In the current study, the group of DEGs described by GO term ‘oxidation reduction’ was the most significantly enriched one (Additional file 5). This group contained several genes associated with hypertension and some of them (*Cyp1a1,* cytochrome P450, family 1, subfamily a, polypeptide 1*; Cyp4f1,* cytochrome P450, family 4, subfamily f, polypeptide 1; *Nos3,* nitric oxide synthase 3, endothelial cell; *Nox4,* NADPH oxidase 4, *Sod3,* superoxide dismutase 3, extracellular) are known as involved in modulation of vascular tone and renal tubular function.

*Cyp1a1* knockout mice are hypertensive. Cyp1a1 metabolizes omega-3 polyunsaturated fatty acids to vasodilators and the loss of these vasodilators may lead to increases in BP [14]. CYP1A1 contributes to eNOS (same as *Nos3*) activation, nitric oxide (NO) bioavailability, and NO-dependent BP regulation [15]. So, the decreased level of *Cyp1a1* transcription in ISIAH renal medulla suggests its contribution to hypertension development in ISIAH rats.

CYP4F1 was characterized as the most critical 4 F isoform involved in the production of 20-hydroxyeicosatetraenoic acid (20-HETE), a potent eicosanoid that modulates vascular tone and renal tubular function [16]. 20-HETE has been shown to play a complex role in BP regulation. In the kidney tubules, 20-HETE inhibits sodium reabsorption and promotes natriuresis, thus, contributing to antihypertensive mechanisms. In contrast, in the microvasculature, 20-HETE plays a pressor role by sensitizing smooth muscle cells to constrictor stimuli and increasing vascular myogenic tone, and by acting on the endothelium to further promote endothelial dysfunction and endothelial activation. In addition, 20-HETE induces endothelial angiotensin-converting enzyme, thus, setting forth a potential feed forward prohypertensive mechanism by stimulating the renin-angiotensin-aldosterone system (RAAS) [17].

Nox4 is a hydrogen peroxide-producing NADPH oxidase isoform highly expressed in the kidney. NAD(P)H oxidase was recognized as the major source of reactive oxygen species (ROS) contributing to salt-induced hypertension [18]. Dahl salt-sensitive hypertensive rats with knockout of Nox4 exhibited a reduced renal injury and attenuated BP response to high salt [19]. Thus, we may suggest that the increased transcription of *Nox4* in the renal medulla of ISIAH rats may contribute to increased rate of ROS production and hypertension development.

Superoxide dismutases (SODs) convert superoxide to hydrogen peroxide, which is then removed by glutathione peroxidase or catalase [20]. The oxidative stress appears to be a common feature of hypertensive disorders from diverse origins [21], and it has been long known that oxidative stress induces or enhances the activity of SODs [22]. Thus, the enhanced expression of *Sod3* provides a rationale to suggest the presence of the oxidative stress in the renal medulla of ISIAH rats which may require the enhanced activity of SOD3 to prevent the formation of highly aggressive ROS.

*Nos3* is known as related to the diversity of biological processes. In the current research, *Nos3* was related to GO term groups such as regulation of BP, oxidation reduction, response to stress and to steroid hormone stimulus, regulation of calcium and sodium ion transport, and angiogenesis (Additional file 5). Endothelial nitric oxide synthase (eNOS; NOS3) is physiologically important for vascular homeostasis [23]. It is expressed predominantly in the endothelium of blood vessels where it catalyzes the production of NO. NO regulates vascular tone and local blood flow, platelet aggregation and adhesion, and leukocyte-endothelial cell interactions. Abnormalities in NO production by the vascular endothelium result in endothelial dysfunction, which occurs in hypertension, diabetes, aging, and as a prelude to atherosclerosis [24].

*Nos3* transcription was found to be reduced in the renal medulla of ISIAH rats. The reduced eNOS expression was earlier reported for different models of hypertension: spontaneously hypertensive rats (SHR) [25], Dahl salt-sensitive rats [26], two-kidney, one-clip hypertensive rats [27]. The reduced level of eNOS and NO production in the renal medulla is associated with a reduction in medullary blood flow and have considerable importance in sodium and water homeostasis and the long-term control of arterial pressure [27–29]. Recently, the reduced renal blood flow measured by magnetic resonance angiography was reported in ISIAH rats as compared to normotensive Wistar rats [30]. So, the reduced transcription of the *Nos3* in the renal medulla of ISIAH rats may be considered as a key feature possibly leading to the medullary blood flow reduction. This, and the decreased NO availability in serum of ISIAH rats and its negative correlation with the BP reported earlier [31], might indicate the impact of this mechanism on stress-sensitive hypertension development.

One more gene which may participate in regulation of NO availability in the renal medulla of ISIAH rats is *Ephx2* encoding the soluble epoxide hydrolase (sEH). In the current study, *Ephx2* was one of the DEGs associated with hypertension and showing the highest differences in expression in ISIAH and WAG renal medulla. sEH metabolizes the epoxyeicosatrienoic acids (EETs) having antihypertensive properties. EETs also possess anti-inflammatory actions that could protect the kidney vasculature from injury during renal and cardiovascular diseases [32]. It was shown that P450 eicosanoids are vasodilatory, largely through their ability to activate eNOS and NO release [33]. sEH is a main effector of angiotensin II-induced hypertension [34]. *Ephx2* was also recognized as a gatekeeper gene contributing to programmed hypertension [35]. In the current study, the PLS regression method, which is commonly used for biomarker selection in metabolomic [36] and gene expression [37] studies, reckoned *Ephx2* as one of the genes making the most significant contribution to the inter-strain differences. All this and the increased transcription of *Ephx2* found earlier in hypothalamus [38] and renal cortex [9] of ISIAH rats prompt us to consider *Ephx2* as a key candidate for further studies of the mechanisms underlying the stress-sensitive hypertension in ISIAH rats. The sEH was already considered as a suitable target for pharmaceutical intervention in the hypertension treatment [39].

The other groups of DEGs, being among the most significantly enriched and described by GO terms ‘response to external stimulus’, ‘response to hormone (endogenous) stimulus’, ‘response to stress’, and ‘homeostatic process’, provide the molecular basis for integrated responses to homeostasis disturbances in the renal medulla of the ISIAH rats (Additional file 5). Most DEGs associated with BP regulation (*Adipoq, Adra1b, Agtr1a, Aqp2, Ephx2, Guca2b, Mif, Nos3, P2rx4, Tacr3*) were related to these groups. As it may be seen from the following discussion, some of these genes may contribute to vascular tone and renal blood flow regulation.

Adiponectin (*Adipoq* gene) stimulates production of nitric oxide in vascular endothelial cells [40] and possess anti-atherogenic properties [41]. The increased expression of *Adipoq* in renal medulla of ISIAH rats may be protective against the reduction in medullary blood flow.

*Agtr1a* (angiotensin II receptor, type 1a) encodes the receptor for angiotensin II, which is the main effector of RAAS [42]. AT1A receptors expressed on the renal vasculature and/or renal tubular epithelia play a critical role in sodium and volume homeostasis [43]. AGTR1a is required for mineralocorticoid receptor stimulation to induce vascular remodeling, inflammation and endothelial dysfunction [44]. It was shown that in the euvolemic state the mean arterial pressure was significantly lower in the AT1A receptor-deficient mice compared with wild-type controls [45]. So, the reduced transcription of *Agtr1a* in ISIAH renal medulla may be considered as a compensatory mechanism against the complications mentioned above.

The alpha1B-adrenoceptors (*Adra1b*) are involved in blood vessel remodeling [46] and mediate the vasoconstrictor actions of the renal sympathetic nerves in rats with renal failure [47, 48]. *Adra1b* is one of the sympathetic nervous system (SNS) components [49]. It is known that increased renal SNS activity reduces urinary sodium and water excretion, renal blood flow and glomerular filtration rate [49]. Thus, the reduced transcription of *Adra1b* in ISIAH renal medulla may be considered as a protective mechanism against the excessive effects of SNS activation reported earlier in ISIAH rats [50].

One more DEG, which may cause the differences in SNS activity in ISIAH and WAG rats is *Comt* (catechol-O-methyltransferase), which encodes the enzyme involved in the degradation of catecholamines. The inhibition of COMT induces dopamine-dependent natriuresis [51]. The *Comt*-gene-disrupted mice were resistant to salt-induced hypertension [52]. So, the decreased expression of *Comt* in the renal medulla of ISIAH rats may lead to increase in renal dopaminergic effects and sodium excretion, and may be considered as an adaptive or protective mechanism for control of hypertension development in ISIAH rats. Earlier, the significantly decreased transcription of *Comt* was also detected in kidney of 6-month old ISIAH rats [53].

MIF (Macrophage migration inhibitory factor) possesses the ability to directly regulate the immunosuppressive actions of glucocorticoids and thus plays a critical role in the host control of inflammation and immunity [54]. MIF is a key factor in atherogenesis [55, 56]. The upregulation of the podocyte-expressed MIF induces an injury of podocytes and accelerates the progression of glomerulosclerosis [57]. In ISIAH rats, the upregulation of *Mif* gene was found both in renal cortex [9] and in renal medulla (the current study), and possibly may contribute to development of atherosclerosis as well as to glomerulo- and medullary sclerosis histologically determined earlier [7, 8].

The changes in expression of DEGs associated with renal hypertension (*Agtr1a, Fn1, Gja1, Lama2, Mmp2, Mmp9, Nos3*) may also be suggested to have a significant impact on disease development in ISIAH rats (Table 2).

Fibronectin 1 is a glycoprotein involved in cell–matrix and cell–cell adhesion [58]. Fibronectin 1 may be induced by different agents including angiotensin II [59] and aldosterone [44]. Both of these stimuli may lead to vascular remodeling and vascular inflammation [44, 60]. So, the reduction in *Fn1* expression may be directed against the excessive vascular complications development in ISIAH renal medulla. It is worth also to mention that almost all (33 gene out of 37) of the multiple DEGs related to GO terms ‘cell adhesion’ and ‘cell–matrix adhesion’ were downregulated in ISIAH renal medulla (Additional file 5), and it is of high probability that those changes, at least in a part, are directed towards the prevention of vascular complications.

The protein encoded by *Gja1* (or *Cx43*, connexin 43) is a component of gap junctions, which permit the passage of ions and chemical mediators from cell to cell [61]. The expression of connexins in renal arterioles is believed to have a profound impact on conducted responses, regulation of arteriolar tonus and renal blood flow. Besides, the cell-to-cell communication mediated by Cx43 channels may contribute to regulating the elasticity of the vascular wall [62]. However, no evidence for an increased abundance of Cx43 in renal arterioles of SHR when compared with normotensive counterparts was found [63]. In the ISIAH renal medulla the increased transcriptional level of *Gja1* was detected. Earlier a differential regulation of aortic Cx43 in different models of hypertension was reported [62], and this may also be true for kidney. This may be one of the features distinguishing the mechanism of hypertension development in ISIAH rats from that in SHR.

LAMA2 is an extracellular matrix protein [64] involved in regulation of cell adhesion. Matrix metalloproteinases (MMP2 and MMP9) belong to a family of metalloendopeptidases that cleave the protein components of the extracellular matrix and thereby play a central role in tissue remodeling [65]. MMP2 (*Mmp2*) and MMP9 (*Mmp9*) are involved in the vascular smooth muscle cell activation and neointimal formation that characterize arterial tissue remodeling after injury [66]. The overexpression of MMP9 and MMP2 have been observed within plaques [67]. The elevated urine values of MMP-9 was recognized as a marker of atherosclerotic disease [68], and, alternatively, the loss of MMP9 reduces atherosclerotic burden [69]. MMP-2 also contributes to the development of atherosclerosis [70]. Taking that into consideration, the decrease in *Mmp2* and *Mmp9* expression may be considered as protective against vascular remodeling in ISIAH renal medulla.

So, in ISIAH renal medulla, several DEGs associated with renal hypertension are related to cell–matrix and cell–cell adhesion processes. It is known that in hypertension the increase in arterial stretch stimulates vessel thickening to normalize the tensile forces. This process requires modification of the extracellular matrix and of cell–matrix interactions [71]. The results of the current study are in a good agreement with this conception and highlighted the major DEGs contributing to the modification of the extracellular matrix and of cell–matrix interactions in renal medulla of rats with stress-sensitive hypertension.

Multiple genes with the changed level of transcription related to ion transport and to regulation of ion transport were found in the renal medulla of ISIAH rats. Many of these genes should be essential for maintaining the sodium and water balance in hypertensive state. However, it is not possible to discuss the role of all the DEGs contributing to these processes within the current paper. We just would like to mention that all the processes including such a basic one as sodium and water balance may undergo the strain-specific regulation. This may be seen, for example, in the case of the *Aqp2* being regulated by vasopressin [72] and playing a role in body water balance [73]. Mice with reduced expression of *Aqp2* have severe polyuria and very low urine osmolality [74]. *Aqp2* transcription was reduced in renal medulla of ISIAH rats. The reduced AQP2 in the renal medulla was reported earlier in the clipped kidney of rats with experimental two-kidney, one clip hypertension [75] and in Milan hypertensive rats [76]. However, the medullary expression of AQP2 protein was increased in DOCA-salt hypertensive rats [77] and in SHR [78]. The existence of the strain-specific differences in the molecular mechanisms of the hypertension development points out the importance of the search for the common features contributing to disease manifestation which may be potentially used as new pharmacological targets.

## Conclusions

The current study helped to identify a number of DEGs specifying the function of renal medulla in ISIAH rats being a model of the stress-sensitive arterial hypertension. The genes already known as associated with hypertension development were mostly discussed. However, the other genes also may have indirect influence on disease development and maintenance. The discussion demonstrated that the changes in expression of many genes may be considered as protective or adaptive and directed toward the integration and coordination of the general homeostasis of the organism. Several DEGs, which may modulate the renal medulla blood flow were detected. The reduced transcription of the *Nos3* points to the possible reduction of the blood flow in the renal medulla of ISIAH rats.

The generated data may be useful for comparison with those from different models of hypertension and for identifying the common molecular determinants contributing to disease manifestation which may be potentially used as new pharmacological targets. Based on discussion, *Ephx2* seems to be a promising candidate for further studies of its potential as therapeutic target.

## Methods

### Animals

The hypertensive ISIAH/Icgn and normotensive WAG/GSto-Icgn rat strains were used in the current study. Rats were bred in the Center for Genetic Resources of Laboratory Animals at the Institute of Cytology and Genetics, Siberian Branch of the Russian Academy of Sciences, (Novosibirsk, Russia, RFMEFI61914X0005 and RFMEFI62114X0010). All rats were kept in standard laboratory conditions and had free access to food and water. The 3-month old ISIAH, and WAG male rats were used in RNA-seq experiments. Each group of experimental animals contained 3 rats. Rats were individually caged one week before the measurement of the systolic arterial BP, which was measured indirectly by the tail-cuff method as it was described earlier [9]. The mean systolic arterial BP was 171.7 ± 1.22 mmHg in the experimental group of ISIAH males and 116.33 ± 1.86 mmHg in WAG. Six days after BP measurement, rats were decapitated and their kidneys were rapidly removed and sectioned. The samples of renal medulla were stored in RNA Later (Qiagen, Chatsworth, CA) at −70 °C until use. The animal experiments were conducted with approval of the Institute’s Animal Care and Use Committee.

### RNA-seq analysis

The samples of renal medulla were sent to JSC Genoanalytica (Moscow, Russia), where the technological part of the RNA-seq analysis was conducted as it was described earlier {9}. Dynabeads mRNA Purification Kit (Ambion, USA) was used for mRNA extraction and NEBNext mRNA Library Prep Reagent Set for Illumina (NEB, USA) was used for cDNA libraries construction. All kits were used according to the manufacturer’s protocol. cDNA libraries were sequenced using a HiSeq1500 Sequencing System (Illumina Sequencing, San Diego, USA) in a single end mode with a read length of 50 bases. Three biological replicates were analyzed for each rat strain. The sequencing data were subjected to adapter trimming and low-quality sequence removal and mapped to the rat reference genome (RGSC Rnor_5.0, rn5) with the use of Tophat2 [79]. Quality metrics of the mapped libraries (Additional file 7) were collected using the Picard ‘CollectRnaSeqMetrics’ tool (http://broadinstitute.github.io/picard/). The differential expression analysis was performed using the Cufflinks workflow [80]. A gene was defined as being expressed if it matched the Cufflinks criteria on suitability for statistical testing (test status ‘OK’). Genes with a Benjamini-Hochberg adjusted p-value (q-value) <0.05 were considered to be differentially expressed. Heatmap of the genes differentially expressed (q value < 0.01) in the renal medulla of the ISIAH and WAG rats was built using the core functions of R statistical language (https://cran.r-project.org/); a hierarchical ‘complete linkage’ clustering by Euclidean distance was used to construct the dendrograms. The raw RNA-Seq data are available at the NCBI Short Read Archive database under the Accession number: PRJNA299102.

### Functional annotation

The DEGs were functionally annotated in DAVID (The Database for Annotation, Visualization and Integrated Discovery) (http://david.abcc.ncifcrf.gov/) [81, 82] with the use of *Rattus norvegicus* genome as the background list for the over-representation analysis. The significantly enriched biological processes (p < 0.05) were identified using the Gene Ontology option. The most significant to the data set pathways were identified in Kyoto Encyclopedia of Genes and Genomes Pathway Database (KEGG, http://www.genome.jp/kegg/). The annotation of DEGs in the Rat Genome Database Disease Portals (RGD, http://rgd.mcw.edu/wg/portals?100) was used to detect the genes related to arterial hypertension and renal diseases. The DEGs encoding the transcription factors were detected using the atlas of combinatorial transcriptional regulation in mouse and man [83], GenBank (http://www.ncbi.nlm.nih.gov/gene/), and Panther classification system (http://www.pantherdb.org/) [84].

### Quantitative real-time PCR (qPCR)

To estimate the relative amount of target mRNA, qPCR analysis was performed on the renal medulla samples from ISIAH (n = 5) and WAG (n = 5) male rats aged as 3-month old. TRI reagent (Molecular research center, USA) was used for the extraction of the total RNA, and DNase I (Promega, USA) treatment was performed to remove the residual genomic DNA. The kits were used according to the manufacturer’s recommendations.

The protocol for the reverse transcription was the following: the reaction was carried out with 40 units of MoMLV (Vektor-Best, Russia) in 50 μl of reaction mixture, which contained reverse transcription (RT) buffer, 0.4 mM dNTPs, 0.25 nmol of random nonanucleotide primers (Biosan, Russia), and 3 μg of total RNA. The cDNA synthesis was performed at 37 °C (1 h), 42 °C (30 min), and 50 °C (10 min) with the following inactivation of the enzyme by heating at 75 °C for 5 min.

The reaction volume for qPCR was 20 μl. It contained a master mix with SYBR Green, 0.15 mM of each forward and reverse primers, 1 unit of HotStart Taq polymerase (Vektor-Best, Russia), and the cDNA template. The *Rpl30* (ribosomal protein L30) stably expressed in different tissues from ISIAH and WAG rats was used as a reference gene. The primers sequences and characteristics are given in Additional file 8.

qPCR was performed in an iCycler iQ4 Real-Time PCR Detection System (Bio-Rad Laboratories, USA) using the protocol described earlier [9]. Relative transcript levels were determined by standard-curve quantitation method [85]. The mixture of the aliquots from all synthesized cDNA samples was used as a standard cDNA for calibration curves plotting. The value obtained for the target gene was normalized against the value for reference gene and then the relative mRNA abundance was calculated as a ratio of the normalized mRNA level in the experimental ISIAH samples to the normalized mRNA level in the samples from WAG rats. A value of 1 was assigned to the normalized mRNA level obtained for the samples from control WAG rats.

### Statistical methods

Mann-Whitney U-test (Statistica v.8.0, Statsoft, USA) was used for calculations of the statistical significance for qPCR data. Statistical significance was set at p < 0.05. The data were expressed as M ± S.E.M. (means and their standard errors).

The acquired RNA-seq data (FPKM values) were log transformed, centered and normalized to run the partial-least squares discriminant analysis (PLS-DA). First, the scaling of the data sets was performed using principal coordinates method based on Euclidean metric distances. Then, the pattern of co-variation for linear combinations between two blocks of variables [86] was explored to get the separation of groups in PLS-DA. PLS-DA and the following calculation of Pearson correlation coefficients were performed to find a set of variables (expressed genes) that maximize the covariance between gene expression in ISIAH and WAG rat strains and fixed dummy matrix representing group membership [86] for WAG and ISIAH rats, correspondingly. As a result of these procedures the PLS-DA Axes maximizing the distances between hypertensive and normotensive rats were constructed, the correlation between gene expression and PLS-DA Axis 1 was calculated, and the genes contributing the most to differences between ISIAH and WAG transcriptomes were revealed.

## Additional files

Additional file 1: Genes differentially expressed in renal medulla of hypertensive ISIAH and normotensive control WAG rats. (XLS 197 kb)
Additional file 2: Heatmap of the differentially expressed genes in the renal medulla of the ISIAH and WAG rats. (PDF 50 kb)
Additional file 3: Genes with detected expression in renal medulla of only one rat strain. (DOC 51 kb)
Additional file 4: Functional annotation of differentially expressed genes (DEGs) found in 3-month old ISIAH and WAG renal medulla. (XLS 95 kb)
Additional file 5: Differentially expressed genes related to Gene Ontlogy (GO) terms for biological processes in the renal medulla of 3-month old ISIAH and WAG male rats. (XLS 160 kb)
Additional file 6: Metabolic pathways enriched with genes differentially expressed in ISIAH and WAG renal medulla. (XLS 41 kb)
Additional file 7: The summary statistics for the sequenced libraries from renal medulla of ISIAH and WAG rats. (XLS 17 kb)
Additional file 8: Primers used in real-time PCR. (DOC 33 kb)

## Abbreviations

20-HETE: 20-hydroxyeicosatetraenoic acid
BP: Blood pressure
DAVID: Database for Annotation, Visualization and Integrated Discovery
DEGs: Differentially expressed genes
EETs: Epoxyeicosatrienoic acids
eNOS or NOS3: endothelial nitric oxide synthase
FPKM: Fragments per kilobase of transcript per million mapped reads
GO: Gene Ontology
ISIAH: Inherited Stress-Induced Arterial Hypertension
KEGG: Kyoto Encyclopedia of Genes and Genomes Pathway Database
NO: Nitric oxide
PLS-DA: Partial-least squares discriminant analysis
qPCR: quantitative real time polymerase chain reaction
RAAS: Renin-angiotensin-aldosterone system
RGD: Rat Genome Database
RNA-seq: RNA sequencing
ROS: Reactive oxygen species
SABP: Systolic arterial blood pressure
sEH: soluble epoxide hydrolase
SHR: Spontaneously hypertensive rats
SNS: Sympathetic nervous system
SODs: Superoxide dismutases
WAG: Wistar Albino Glaxo rats
