# Supplementary material for: The gene-expression profile of renal medulla in ISIAH rats with inherited stress-induced arterial hypertension
Source: BMC Genet. 2016 Dec 22;17(Suppl 3):151. doi: 10.1186/s12863-016-0462-6 (PMC5249016; doi:10.1186/s12863-016-0462-6)
Supplement: Additional file 2: — Heatmap of the differentially expressed genes in the renal medulla of the ISIAH and WAG rats. (PDF 50 kb) [file 12863_2016_462_MOESM2_ESM.pdf]

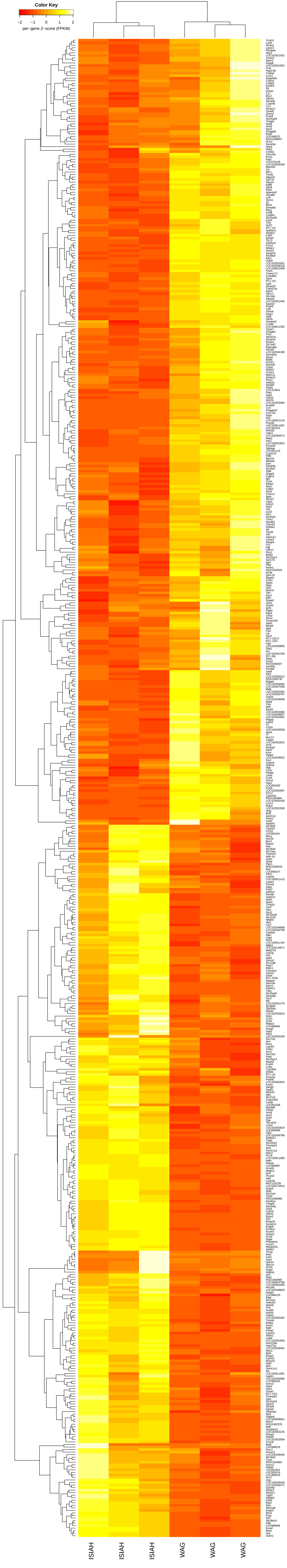

Additional file 2. Heatmap of the differentially expressed genes in the renal medulla of the ISIAH and WAG rats. Yellow depicts genes that were up−regulated in a sample, and red depicts those that were down−regulated.
