# Supplementary material for: The gene-expression profile of renal medulla in ISIAH rats with inherited stress-induced arterial hypertension
Source: BMC Genet. 2016 Dec 22;17(Suppl 3):151. doi: 10.1186/s12863-016-0462-6 (PMC5249016; doi:10.1186/s12863-016-0462-6)
Supplement: Additional file 3: — Genes with detected expression in renal medulla of only one rat strain. (DOC 51 kb) [file 12863_2016_462_MOESM3_ESM.doc]

Additional file 3. Genes with detected expression in renal medulla of only one rat strain

| Gene symbol | NCBI gene ID | Value, FPKM | | Gene name | q_value |
| --- | --- | --- | --- | --- | --- |
|  |  | ISIAH | WAG |  |  |
| *Hand2* | 64637 | 3.04 | 0 | heart and neural crest derivatives expressed 2 | 0.026 |
| *LOC102555985* | 102555985 | 2.26 | 0 | uncharacterized | 0.002 |
| *LOC688516* | 688516 | 1.50 | 0 | similar to L-lactate dehydrogenase A chain (LDH-A) (LDH muscle subunit) (LDH-M) | 0.002 |
| *Retn** | 246250 | 3.06 | 0 | resistin | 0.002 |
| *Rpl38-ps1* | 690833 | 2.11 | 0 | ribosomal protein L38, pseudogene 1 | 0.026 |
| *Cyp2c24* | 499353 | 0 | 17.6 | cytochrome P450, family 2, subfamily c, polypeptide 24 | 0.002 |
| *LOC100362965* | 100362965 | 0 | 3.8 | SNRPN upstream reading frame protein-like | 0.02 |
| *LOC102546857* | 102546857 | 0 | 2.7 | NA | 0.002 |
| *LOC102553584* | 102553584 | 0 | 1.9 | uncharacterized | 0.002 |
| *Sfta2* | 415052 | 0 | 8.7 | surfactant associated 2 | 0.002 |
| *Slpil2* | 408229 | 0 | 1.6 | antileukoproteinase-like 2 | 0.009 |

* - genes associated with hypertension (according to genes annotations in Rat Genome Database, http://rgd.mcw.edu/). ISIAH and WAG – rat strains used in the study.

FPKM (fragments per kilobase of transcript per million mapped reads.
