# Supplementary material for: The gene-expression profile of renal medulla in ISIAH rats with inherited stress-induced arterial hypertension
Source: BMC Genet. 2016 Dec 22;17(Suppl 3):151. doi: 10.1186/s12863-016-0462-6 (PMC5249016; doi:10.1186/s12863-016-0462-6)
Supplement: Additional file 8: — Primers used in real-time PCR. (DOC 33 kb) [file 12863_2016_462_MOESM8_ESM.doc]

**Additional file 8.**

Primers used in real-time PCR

| Gene | Primers, 5′-->3′ | T_anneal_,  ^o^C | T_acq_,  ^o^C | Length of  PCR fragment, bp |
| --- | --- | --- | --- | --- |
| *Comt* | F: CTTGACCACTGGAAAGACCG  R: CGATGACGTTGTCAGCTAGGA | 61 | 84 | 100 |
| *Ephx2* | F: TTTCTTGGAGGTACCAGATCC  R: CAGTCATGGCCAATGAACAC | 62 | 84 | 193 |
| *Acsm3* | F: CTGCCTACCGGATGCTT  R: CACAGATCAGCACCGTTTC | 63 | 83 | 174 |
| *Adra1b* | F: CCAAAACCTTGGGCATTGTA  R: TAGATGATGGGATTGAGGCA | 64 | 87 | 166 |
| *Rcan1* | F: TGGCAAACGGTGATGTCTTC  R: TGTAAAGTCTGGGCAAAGTACA | 63 | 83 | 220 |
| *Rpl30* | F: CATCTTGGCGTCTGATCTTG  R: TCAGAGTCTGTTTGTACCCC | 61-64* | 84 | 143 |

*T*_anneal_ – the annealing temperature used in experiment, *T*_acq_ - the temperature of fluorescence signal acquisition. *– the range of appropriate annealing temperatures for reference gene. F – forward primer; R - reverse primer.
